# Air classification of copper granules from recycled electrical cables using a zig-zag separator

**DOI:** 10.1038/s41598-026-42336-y

**Published:** 2026-03-04

**Authors:** Piotr Madej, Radosław Zybała, Agata Rządzka-Madej, Maciej Ryłko

**Affiliations:** 1 Łukasiewicz Research Network–Institute of Non-Ferrous Metals, ul. Sowińskiego 5, Gliwice, 44-100 Poland; 2 Mercury HM sp. z o.o. sp.k., ul. Konstantego Ildefonsa Gałczyńskiego 6, Bielsko-Biała, 43–300 Poland

**Keywords:** CFD numerical analysis, Recycling, Power cables, Cable scrap, Copper recovery, Energy science and technology, Engineering, Environmental sciences

## Abstract

Recycling copper electrical power cable scrap presents challenges in achieving high purity of the final product, which is typically in the form of copper granules. To obtain copper granules of the highest possible purity, it is necessary to separate impurities, primarily tin-contaminated copper granules (from tinned elements), from pure copper granules. To investigate the feasibility of separating tin-contaminated (tinned) copper using a zig-zag air classifier, numerical CFD analyses of the process were conducted. These simulations were subsequently validated against experimental studies performed with a laboratory-scale zig-zag air classifier. The conducted research demonstrated that the application of a zig-zag air classifier allows to produce copper granules with a final copper content exceeding 99% Cu. A comparison of the numerical CFD separation process analysis results with the actual experimental data revealed that the model’s error is dependent on the morphology of the analyzed granules.

## Introduction

The development of the global economy requires the efficient utilization of primary raw materials and their protection through increased recycling rates of secondary resources.

Since the beginning of the 20th century, the demand for copper has been steadily rising. BHP, a global resources company, estimates that copper demand will increase by approximately 70% between 2021 and 2050^1^.

Alongside iron, copper has played a unique role in the advancement of human civilization. This metal finds its primary applications in electrical equipment (32%), construction (26.2%), infrastructure (17.3%), transport (12.9%), and industry (11.5%)^2^. An additional factor driving the ever-growing demand for this raw material is the progress in research and the development of technological innovations.

Copper is an essential metal for energy storage technologies, particularly for electric vehicles (EVs). Its excellent thermal and electrical conductivity, combined with 100% recyclability, make it an ideal raw material for building a sustainable world^3^.

According to experts from the European Copper Institute, the photovoltaic boom will also lead to a significant increase in copper demand. Over the next 20 years, requirements are expected to reach as much as 87,000 tonnes. This implies that during this period, Poland alone will need approximately 87,000 tonnes of copper for the construction of photovoltaic installations. Electromobility will further drive global copper demand; while a conventional internal combustion engine vehicle requires about 22.3 kg of copper, an electric vehicle can consume up to 53.2 kg^4^.

Current copper products contain a high mass fraction of recycled Cu. According to the United States Geological Survey, 35% of global demand is currently met by recycled copper. Copper can be infinitely processed and reused multiple times without losing its properties. Utilizing secondary raw materials reduces the extraction of primary ores and, consequently, minimizes environmental pollution. Producing 1 Mg of copper through recycling requires 85% less energy than primary production from mineral ores. Recovering copper from waste also allows for the reduction of existing waste volumes and minimizes their environmental impact.

## Construction of power cables and morphology of copper granules obtained from them

**Fig. 1:**
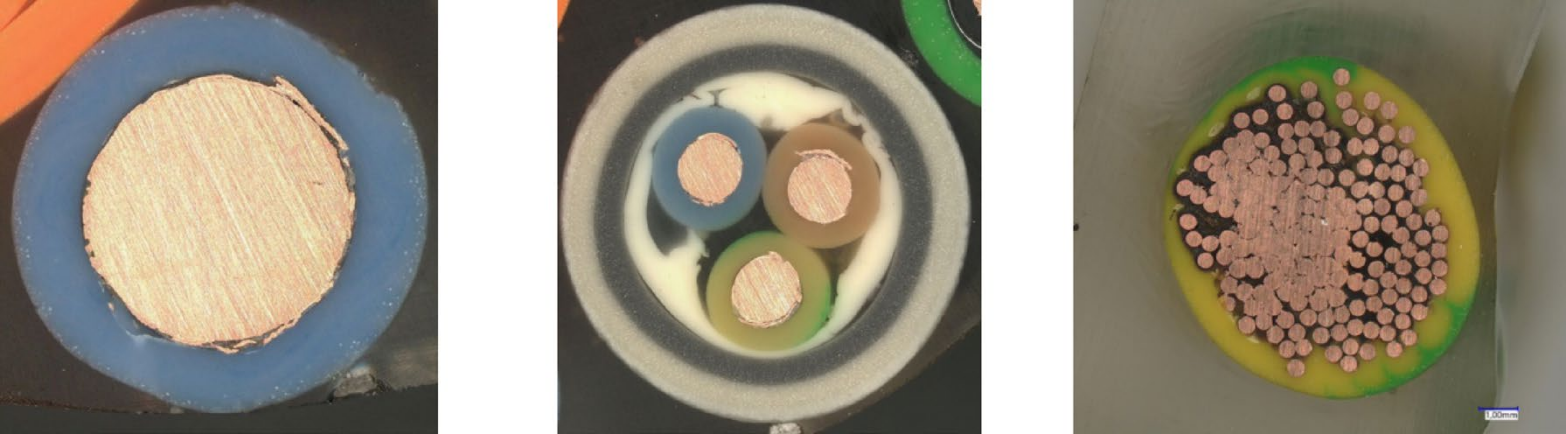
Design of copper cables: (from left) single-core solid conductor cable, three-core cable, and stranded single-core cable.

In simplified terms, electrical cables consist of an outer sheath (insulation), a shield (braiding), inner insulation, and a core (conductor), which serves as the electrical conductor. Cables can be classified as single-core or multi-core (Fig. 1). The cable core is typically made of either aluminum or copper^5^.

**Fig. 2:**
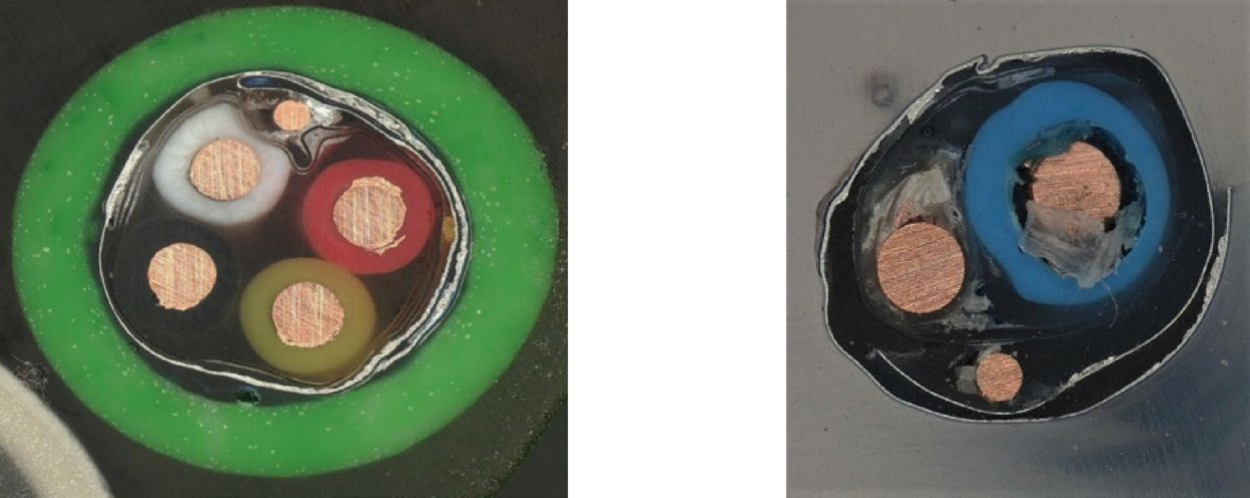
Signal cables with aluminum foil shielding (left) and lead foil shielding (right).

Each conductor (core) may consist of a single solid wire or a stranded wire (so-called “rope”) composed of several to dozens of thin wires with diameters in the tenths of a millimeter. Stranded cables are often tinned to prepare them for assembly in various connectors and plugs. The tin coating provides mechanical protection against damage and prevents oxidation. Additionally, some cable types include structural elements made of steel, aluminum, or lead, which are intended to increase mechanical strength or shield the conductor from external interference (Fig. 2).

Furthermore, the electrical insulation constituting the outer layer protects the entire cable from mechanical damage, atmospheric conditions, and protects the user against electric shock^6^. Cables vary not only in their core structure but also in the material used for insulation. Common materials used for conductor insulation include^7^: polyvinyl chloride (PVC), polyethylene (PE), rubber, and specialized materials such as silicone or polyurethane. In Poland, at least 400,000 tonnes of various types of power cables are produced annually^8^. Consequently, an equivalent mass of power cable scrap enters the recycling stream each year.

Multi-stage processing lines are employed in the recycling technologies of electrical cable and wire scrap. The components of these lines are designed for shredding, milling, and the removal of insulation, plastics, braiding, and steel structural elements, as well as for the recovery of copper. The machinery used in recycling includes shredders, mills, belt conveyors, vibrating tables, magnetic and electrostatic separators, air classifiers, and auxiliary equipment such as dust extraction systems.

Numerous studies have been published regarding the recycling of power cables. In^9^, laboratory tests on the separation of electrical insulation from the primary copper stream were described. A mixture of plastics and copper with a fraction size below 2 mm was subjected to separation using a shaking table and flotation. The achieved purity of the final product was 97% Cu.

In^10^, an eddy-current/electrostatic separator was utilized to recover copper from cable scrap. Furthermore^11^, investigated the possibility of recovering Cu from plastic granulate (resulting from the initial separation of copper and insulation on an air-shaking table) containing up to 15% Cu. Similar research was conducted in^12^, where mechanical methods for copper recovery were examined.

A completely different approach was proposed in^13^, where the possibility of recovering copper from cables at low temperatures (115 K) was explored. The process involved freezing the insulation to make it brittle, followed by its removal through milling. Numerous patents have also been filed, such as CN209766133, CN209249216, and CN204215821, which describe mechanical methods for recovering copper from waste cables.

An example of a technology utilizing exclusively mechanical recycling methods for power cables is the solution developed and implemented under project POIR.01.01.01-00.01-0216/21 at the MERCURY HM company, operating in Bielsko-Biała.

MERCURY HM Sp. z o.o., sp. k. is a company operating in the non-ferrous metal recycling industry, primarily focusing on copper. The enterprise specializes in the sourcing and recovery of non-ferrous metals from power cable scrap using mechanical separation methods. The copper granulates recovered through these operations, characterized by a purity exceeding 99% Cu, are sold to processing plants and smelters, where further processing is carried out using metallurgical methods. The technology implemented at the facility consists of three technological stages:

- Technological Stage no. I – preliminary shredding of cable scrap to a fraction size below and magnetic separation of the copper granulate and insulation mixture to remove magnetic components,
- Technological Stage no. II – secondary grinding to a fraction size below 4.5 mm,
- Technological Stage no. III –separation of high-grade pure copper (Cu) granulate with a minimum purity of 99% Cu from plastic granulate (electrical cable insulation) and tinned copper (CuSn) granulate with a purity of 93–98% Cu.

**Fig. 3:**
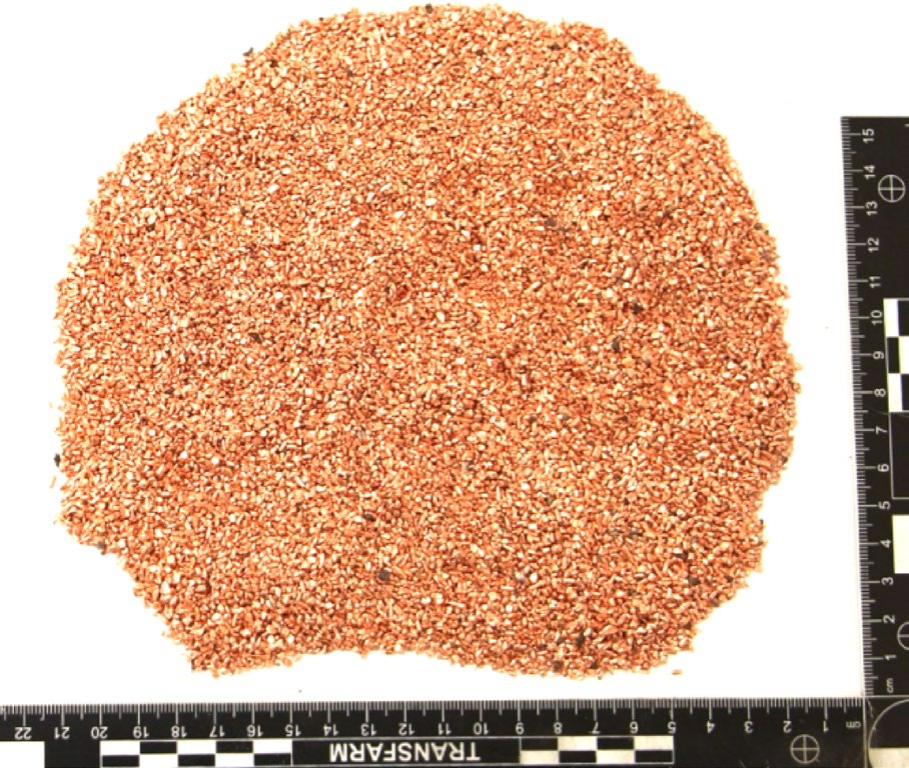
Copper granules with a purity of 97–98% Cu, recovered from a power cable scrap recycling line.

The final product of the recycling process, obtained according to the solution described above, is a copper granulate with a purity ranging from 97 to 98% Cu (Fig. 3), derived from a mixture of power cable scrap.

**Fig. 4:**
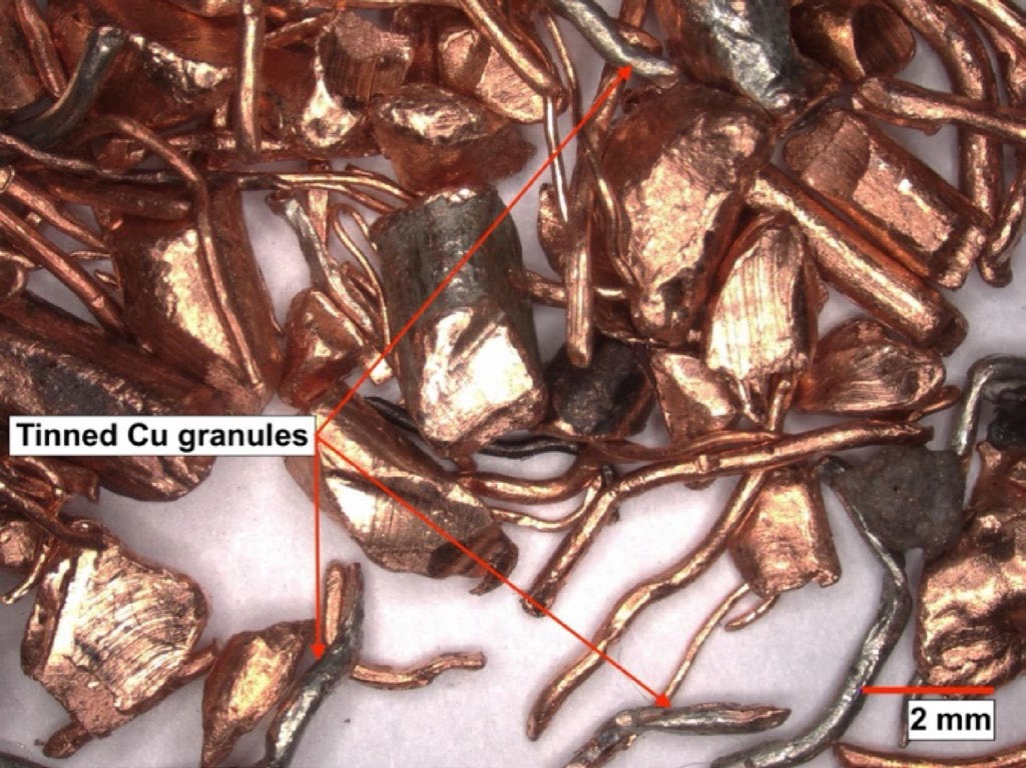
Macro photograph of recycled copper granules (97–98% Cu) from power cable scrap, featuring tin impurities present as a coating on the copper surface.

The impurities identified in the copper granulate recovered from power cable recycling lines consist mainly of copper granules with a surface layer of tin. These are cylindrical elements with a diameter of 0.1–0.2 mm and a length of up to 5 mm (Fig. 4). During the recycling of stranded-core cables, these tinned copper granules report to the final copper product, thereby reducing its quality. Their complete removal using a gravity-air shaking table is impossible. Therefore, this study investigates the separation of tinned copper granules using a zig-zag air classifier (zig-zag separator).

### Design and principle of operation of the zig-zag air separator

**Fig. 5:**
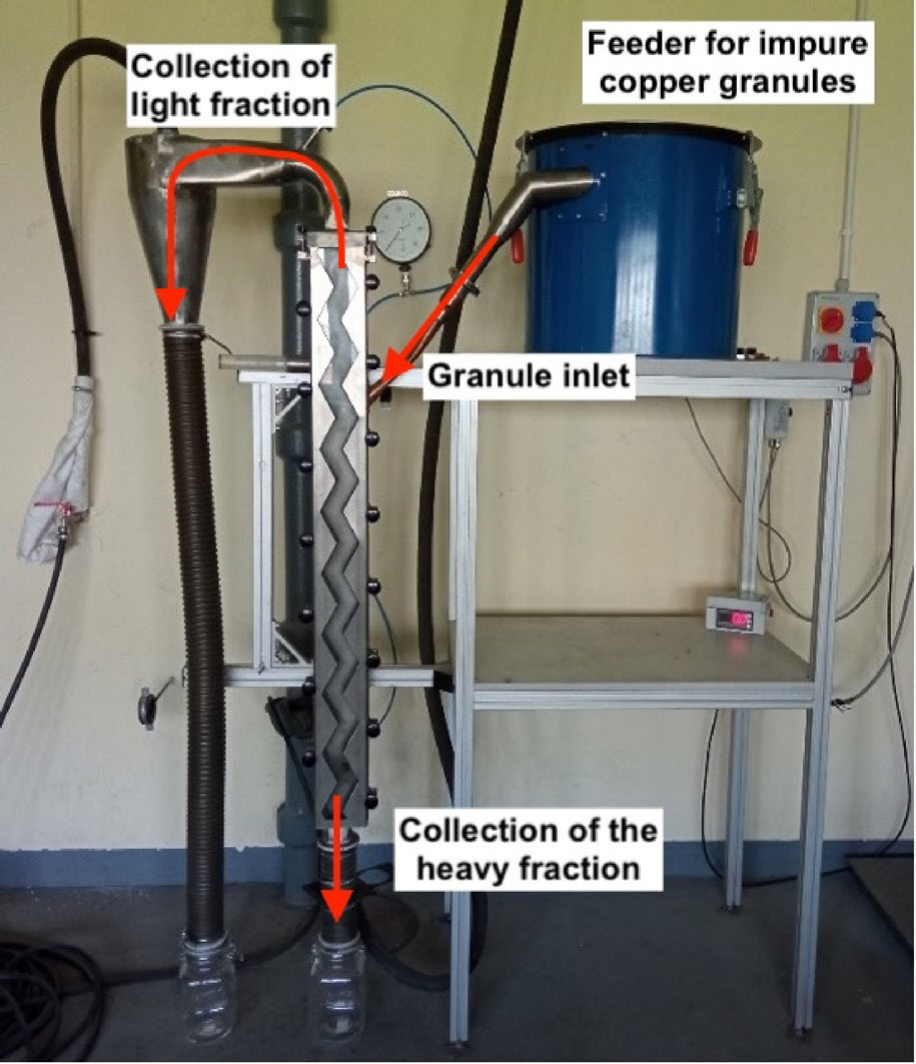
Laboratory zig-zag air classifier.

The separation process in a zig-zag air classifier is based on the difference in the buoyancy and drag forces acting on particles within a gas (air) stream. The zig-zag separator consists of rectangular sections joined at specifically selected angles to form a series of bends, creating a characteristic zig-zag shape (Fig. 5).

Particles introduced into this channel undergo aerodynamic separation. Particles (granules) with a buoyancy force lower than the upward force generated by the flowing air (supplied at the bottom of the channel) are carried by the gas stream toward the upper outlet, forming the light fraction. Conversely, particles where the gravitational force exceeds the aerodynamic lift will fall toward the bottom outlet, forming the heavy fraction.

**Fig. 6:**
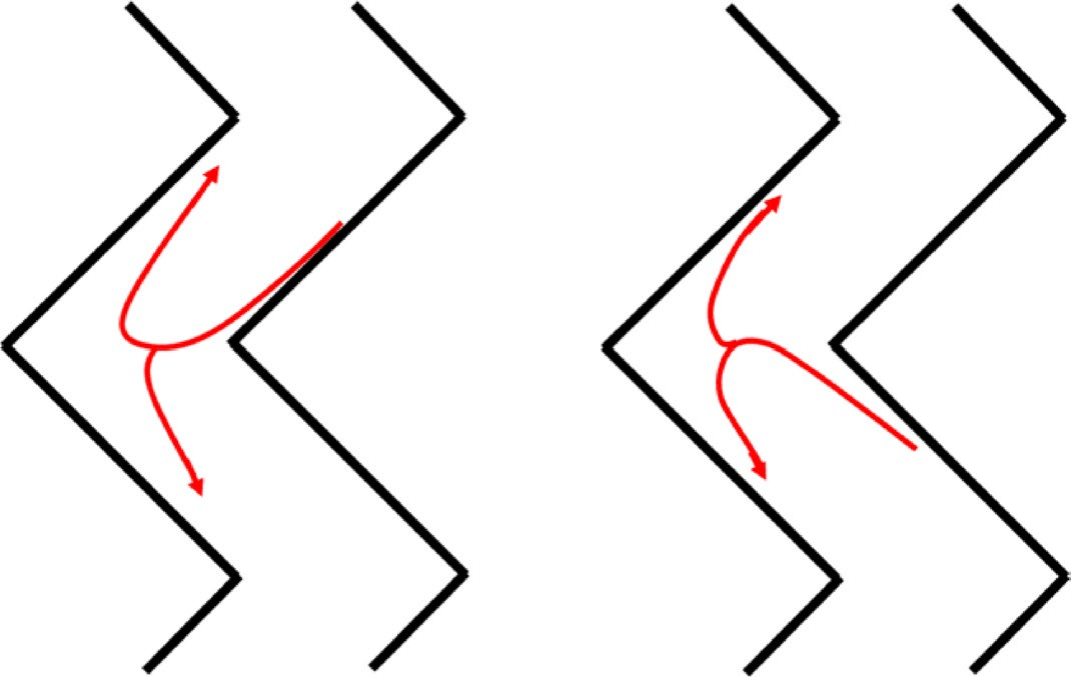
Schematic of particle trajectory variations within airflows: heavy fraction particles exiting the channel in the descending stream (left) and light fraction particles exiting in the ascending stream (right).

Within the individual sections of the device, distinct ascending and descending particle streams occur. Descending streams are observed along the inner walls, while ascending streams form along the outer walls (Fig. 6). During the separation process, particles moving within a given airstream may switch streams at the section junctions or remain within the same flow. The particle trajectories result from the characteristic airflow profile in the channel, which enables the separation of particles based on their mass and aerodynamic properties.

The physical basis of the separation process using a zig-zag classifier is achieving a balance between the gravitational force (Fgravity) and the aerodynamic drag force (Fdrag)^14,15^:

$${\mathrm{F}}_{gravity}={\mathrm{F}}_{drag}$$

This balance is achieved at the terminal velocity (v_terminal_), which can be determined using the Stokes Eqs^14,15^. :

$${\mathrm{v}}_{terminal}=\left(\frac{2\cdot{r}^{2}\cdot{\rho}_{particle}}{9\cdot{\mu}_{air}}\right)$$

where: *r* – is the particle radius, *ρ* – s the particle density, *µ* – is the dynamic viscosity coefficient.

Determining the terminal velocity and the balance of forces within the separator allows for the characterization of particle separation. This separation can be described as a relationship between the forces^14,15^:

The relationship between these forces determines the particle behavior as follows:

- For light particles: F_drag_ > F_gravity_ – upward particle entrainment.
- For heavy particles: F_gravity_ > F_drag_ – downward particle settling.
- Cut point: v_terminal = v_air_ (r equilibrium state).

Both phenomena can be separated by the equilibrium state, where^14,15^:

$${\mathrm{v}}_{terminal}={\mathrm{v}}_{air}$$

at this point, the particles remain suspended within the separator.

The efficiency of the separator is most significantly influenced by the relationship between the „separation sharpness” (a parameter indicating the amount of misclassified particles) and the throughput. In addition to these factors, efficiency is further affected by parameters such as channel geometry, the position of the material inlet into the separator’s working channel, the type of separated particles, and the chemical composition of the feed.

For an ideal separation of identical feed particles, equal masses of heavy and light fractions would be produced. Consequently, at lower velocities, the entire feed forms the heavy fraction, while at higher velocities, it forms the light fraction. However, in reality, achieving such conditions is impossible due to phenomena that disrupt the separation process, such as gas stream velocity gradients within the channel, airflow turbulence, inter-particle interactions, and particle-wall collisions.

## Studies on the separation of copper granules

Laboratory tests of the separation process were conducted using a zig-zag air classifier (Fig. 5) on copper granulates obtained from an industrial power cable recycling line.

The experiments involved feeding 500 g of copper granulate contaminated with tinned granules into the zig-zag separator at specific airflow rates. The tests were performed for four types of granulates at airflow rates of 30 and 50 Nm³/h. The resulting separation products (light and heavy fractions) were weighed to determine their percentage share and then subjected to chemical analysis. The results are presented in Tables 1, 2, 3 and 4.

**Table 1:** Chemical composition of the separation products and the Granulate 1 feed sample.

Component	Granulate 1	30 Nm^3^/h		50 Nm^3^/h	
	%				
	Initial composition	Heavy fraction	Light fraction	Heavy fraction	Light fraction
Tin (Sn)	0.110	0.047	0.140	0.004	0.140
Zinc (Zn)	0.051	0.160	0.057	0.002	0.320
Aluminium (Al)	0.032	0.001	0.002	0.001	0.038
Lead (Pb)	0.005	0.004	0.005	0.18	0.007
Iron (Fe)	0.019	0.001	0.003	0.003	0.093
Copper (Cu)	99.78	99.78	99.78	99.81	99.39

**Table 2:** Chemical composition of the separation products and the Granulate 2 feed sample.

Component	Granulate 2	30 Nm^3^/h		50 Nm^3^/h	
	%				
	Initial composition	Heavy fraction	Light fraction	Heavy fraction	Light fraction
Tin (Sn)	0.018	0.003	0.074	0.001	0.061
Zinc (Zn)	0.37	0.400	0.230	0.094	0.770
Aluminium (Al)	0.21	0.004	0.230	0.002	0.470
Lead (Pb)	0.022	0.035	0.93	0.025	0.110
Iron (Fe)	0.11	0.053	0.210	0.001	0.200
Copper (Cu)	99.27	99.50	99.15	99.88	98.33

**Table 3:** Chemical composition of the separation products and the Granulate 3 feed sample.

Component	Granulate 3	30 Nm^3^/h		50 Nm^3^/h	
	%				
	Initial composition	Heavy fraction	Light fraction	Heavy fraction	Light fraction
Tin (Sn)	0.020	0.023	0.100	0.031	0.082
Zinc (Zn)	0.230	0.210	0.096	0.017	0.094
Aluminium (Al)	0.250	0.300	0.047	0.001	0.170
Lead (Pb)	0.360	0.180	0.053	0.380	0.075
Iron (Fe)	0.015	0.052	0.004	0.001	0.005
Copper (Cu)	99.11	99.22	99.63	99.58	99.57

**Table 4:** Chemical composition of the separation products and the Granulate 4 feed sample.

Component	Granulate 4	30 Nm^3^/h		50 Nm^3^/h	
	%				
	Initial composition	Heavy fraction	Light fraction	Heavy fraction	Light fraction
Tin (Sn)	0.068	0.078	0.041	1.630	0.086
Zinc (Zn)	0.300	0.520	0.042	2.490	0.400
Aluminium (Al)	0.150	0.003	0.300	0.002	0.120
Lead (Pb)	0.039	0.012	0.007	0.990	0.008
Iron (Fe)	0.200	0.011	0.076	0.086	0.150
Copper (Cu)	99.21	99.35	99.52	94.62	99.17

Analyses were performed using optical emission spectrometry (OES) with a Thermo Fisher Scientific 4460 simultaneous emission spectrometer equipped with low-voltage spark excitation. As this analytical technique requires solid alloy samples, the collected granulate was remelted to obtain a uniform ingot for chemical analysis. The analytical surface was prepared by milling using a HERZOG laboratory milling machine. For cathode copper measurements, the Cu_CS analytical program was utilized. The spectrometer calibration was based on a series of certified reference materials for high-purity copper (CS series) produced by Łukasiewicz – IMN. Each analytical surface was excited three times, and the final result was calculated as the mean of these three measurements. The described method was used to determine the content of impurities in the copper, namely: Sn, Zn, Al, Pb, and Fe. The copper content was subsequently determined based on the mass balance (relative to the granulate mass before remelting).

Chemical analyses of the individual fractions after air separation revealed that increasing the airflow rate from 30 to 50 Nm³/h leads to an increase in the purity of the heavy fraction – the primary product of the process.

In the case of Granulate 1 (Table 1), at an airflow rate of 30 Nm³/h, no change in copper content was observed in the separation products compared to the feed material (both heavy and light fractions contained 99.78% Cu). Increasing the airflow rate to 50 Nm³/h resulted in an increase in the copper content of the heavy fraction from 99.78% to 99.81%, with a simultaneous decrease in the Cu content of the light fraction from 99.87% to 99.39%.

For Granulate 2 (Table 2), at an airflow rate of 30 Nm³/h, a slight increase in the copper content of the heavy fraction was observed compared to the feed material (from 99.27% to 99.50% Cu). Increasing the airflow rate to 50 Nm³/h led to a further increase in the copper content of the heavy fraction, reaching 99.88%. The resulting light fraction, regardless of the separation airflow rate, consistently showed a lower Cu content than the heavy fraction—at 99.15% and 98.33%, respectively.

Chemical analyses of the separation products for Granulate 3 showed an increase in the purity of both the heavy and light fractions after separation (Table 3). While the increase in the purity of the heavy fraction at 30 Nm³/h was minor—with the Cu content rising from 99.11% in the feed to 99.22%—the light fraction reached a purity of 99.63%. Increasing the separation airflow rate from 30 to 50 Nm³/h resulted in the purity of the heavy fraction rising to 99.58%, while the light fraction maintained a comparable purity level of 99.57%.

Considering the separation results obtained for Granulate 3, it is evident that the light fraction constitutes the material with higher purity. This occurs because the impurities report to the heavy fraction.

**Fig. 7:**
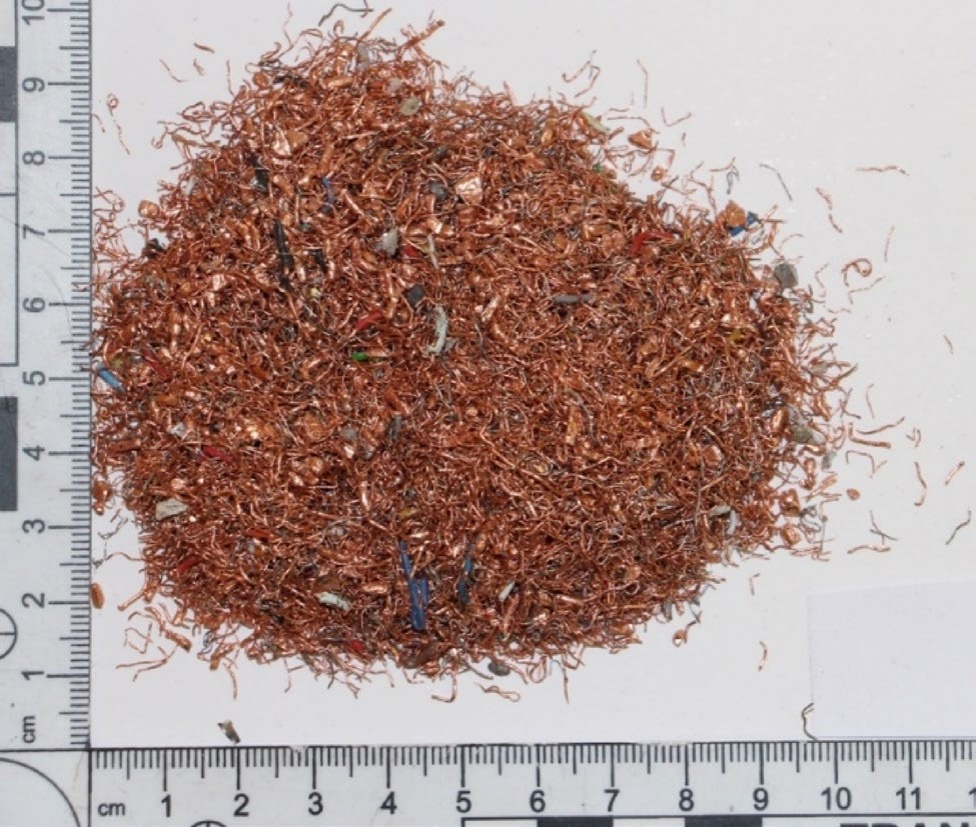
Morphology of Granulate 3.

Figure 7 presents a photograph illustrating the morphology of Granulate 3. It shows that the granulate originates primarily from the processing of stranded wire cables and consists mainly of thin wires. Furthermore, it is characterized by a high volume of non-metallic inclusions (included in the mass balance), as well as metallic inclusions in the form of lead and steel granules (1–2 mm in size), which report to the heavy fraction after separation.

The impurities reducing the purity of the heavy fraction are precisely the Pb and Fe granules introduced with the feed, which report to the heavy fraction, thereby lowering its copper content. Conversely, the clean wires (carried by the airflow) report to the light fraction.

In the case of Granulate 4 (Table 4), the results show that separation yields a higher-purity product at an airflow rate of 30 Nm³/h. Similarly, in this instance, the light fraction is the cleaner product. The granulate possesses a morphology similar to that of Granulate 3, which explains why the light fraction is of higher purity here as well. Increasing the separation airflow rate to 50 Nm³/h does not improve the purity of the resulting separation products.

## Numerical solution approach

Computational Fluid Dynamics (CFD) is a rapidly advancing field of science that emerged in the early 1970s. In recent years, the growth of computational power has enabled the numerical resolution of viscous fluid flow problems in increasingly complex geometries, as well as the simulation of sophisticated phenomena occurring within the fluid volume.

Fluid mechanics, like any other mechanical science, is governed by a series of fundamental laws. However, the equations describing these laws do not fully reflect real-world flows, as they apply only to idealized cases.

In 1904, Ludwig Prandtl simplified the problems of viscous flow analysis by providing an approximate description of boundary layer formation. Nevertheless, describing flows at relatively low velocities remains a significant challenge, where viscous interactions are substantial enough to induce chaotic fluid motion known as turbulent flow^16^.

The earliest CFD calculation methods were limited to the analysis of cases where fluid moved at transonic or supersonic speeds. Further development of numerical methods enabled the resolution of two-dimensional Euler equations, and subsequently, three-dimensional cases. In the mid-1980s, research began on simulating viscous flows by solving the Navier-Stokes equations^17^.

In this article, the motion of copper granulate particles was analyzed using the Lagrangian Discrete Phase Model (DPM). Its primary assumption is a low concentration of the discrete phase (solids) within the continuous phase (air), which allows for its neglect when solving the continuity equations. This means that the discrete phase is tracked as a large group of particles within the fluid flow field. The DPM model utilizes the equations of conservation of mass (Eq. 1) and momentum (Eq. 2)^18–20^.

1 $$\frac{\partial\rho}{\partial t}+\nabla\cdot\left(\rho\mathrm{v}\right)={S}_{DPM}+{S}_{m}$$

where: ρ – denotes density, t – time, v – represents the velocity vector, S_m_ – s the mass source term, S_DPM_ – is the mass source resulting from the discrete particle flow.

2 $$\frac{\partial\rho\mathrm{v}}{\partial t}+\nabla\cdot\left(\rho\mathrm{v}\mathrm{v}\right)=-\nabla p+\nabla\cdot\tau+\rho\mathrm{g}+{\mathrm{F}}_{\mathrm{D}\mathrm{P}\mathrm{M}}+{\mathrm{F}}_{\mathrm{o}\mathrm{t}\mathrm{h}\mathrm{e}\mathrm{r}}$$

where: p – denotes pressure, τ – is the viscous stress tensor, g – represents the gravitational acceleration vector, F_other_ – denotes external body forces, F_DPM_ – represents the body forces exerted by the discrete phase.

The calculation of the discrete phase trajectories is performed by integrating the balance of forces acting on the particles within the Lagrangian reference frame (Eq. 3)^21–23^.

3 $${m}_{p}\frac{d{\mathrm{u}}_{\mathrm{p}}}{dt}={m}_{p}\frac{\mathrm{u}-{\mathrm{u}}_{\mathrm{p}}}{{\tau}_{r}}+{m}_{p}\frac{\mathrm{g}\left({\rho}_{p}-\rho\right)}{{\rho}_{p}}+\mathrm{F}$$

where: m_p_ – the particle mass, u – the fluid phase velocity, u_p_ – the particle velocity, *ρ* - the fluid density, g - the gravitational acceleration vector, *ρ*_*p*_ *–* the density of the particle, F – an additional force; *τ*_*r*_ – the droplet or particle relaxation time^15,16^.

4 $${m}_{p}\frac{\mathrm{u}-{\mathrm{u}}_{\mathrm{p}}}{{\tau}_{r}}$$

where: u – the fluid phase velocity, u_p_ – the particle velocity, *τ*_*r*_ – the droplet or particle relaxation time.

Equation 4 allows for the determination of the fluid drag force. The relaxation time of a droplet or particle (*τ*_*r*_) s described by Eq. 5:

5 $${\tau}_{r}=\frac{{\rho}_{p}{d}_{p}^{3}}{18\mu}\frac{24}{{C}_{d}Re}$$

where: *µ* – the molecular viscosity of the fluid, Re – the relative Reynolds number, *ρ*_*p*_ *–* the density of the particle, d_p_ – the particle size, C_d_ – the drag coefficient.

Wartość relatywnej liczby Reynoldsa jest obliczana z równania 6.

6 $$Re\equiv\frac{\rho{d}_{p}\left|{\mathrm{u}}_{\mathrm{p}}-\mathrm{u}\right|}{\mu}$$

where: g - the gravitational acceleration vector, d_p_ – the particle size, u – the fluid phase velocity, u_p_ – the particle velocity, *µ* – the molecular viscosity of the fluid.

## Boundary conditions

**Fig. 8:**
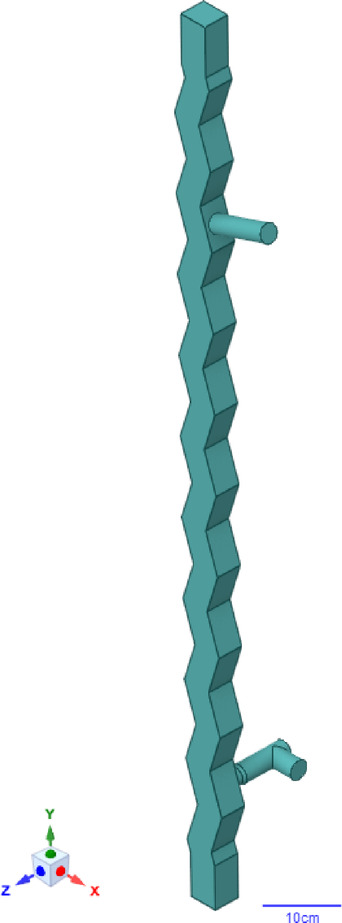
Geometric model of the zig-zag air classifier. The 3D model was developed in SpaceClaim as a fluid volume representing the internal working domain of the separator.

The geometric model of the zig-zag air classifier channel was developed based on the actual separator from the equipment resources of the Metallurgy Centre at the Łukasiewicz Research Network – Institute of Non-Ferrous Metals (Fig. 8).

**Fig. 9:**
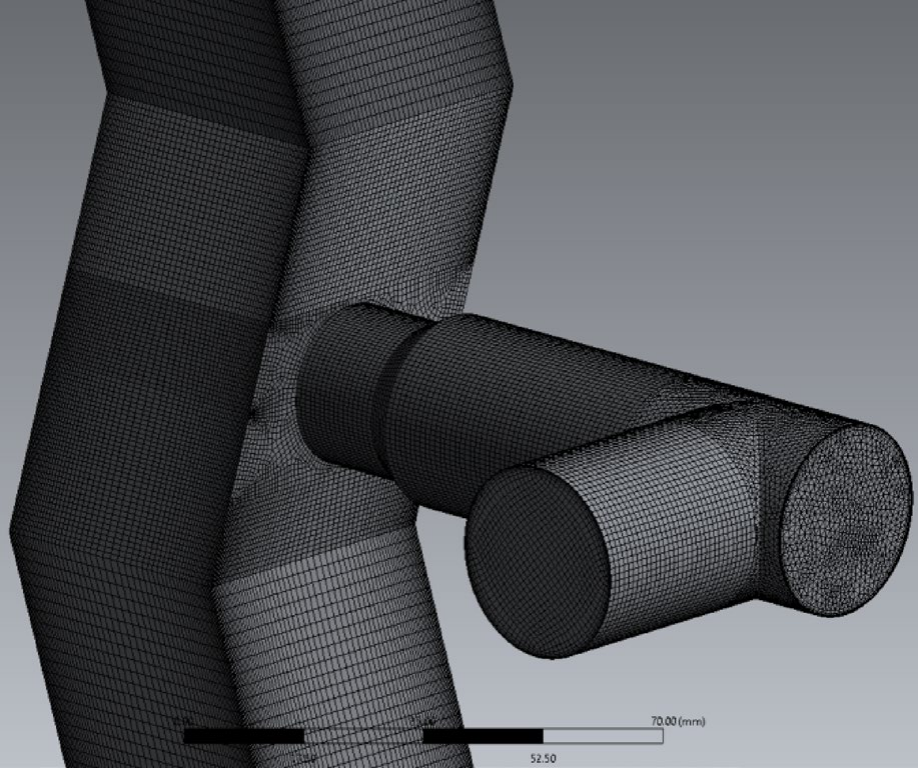
Computational mesh generated on the separator geometry.

The prepared volume was discretized using ANSYS^®^ Meshing into a finite mesh of differential elements (Fig. 9). The model was designed to incorporate the maximum possible number of hexagonal (hex) elements. To achieve this, a multi-component meshing technique was employed (Table 5).

**Table 5:** Computational mesh parameters of the numerical model.

Nodes	6 287 192
Elements	2 710 385
Orthagonal Quality	
Min	0.22
Average	0.94
Skewness	
Max	0.54
Average	0.17

**Fig. 10:**
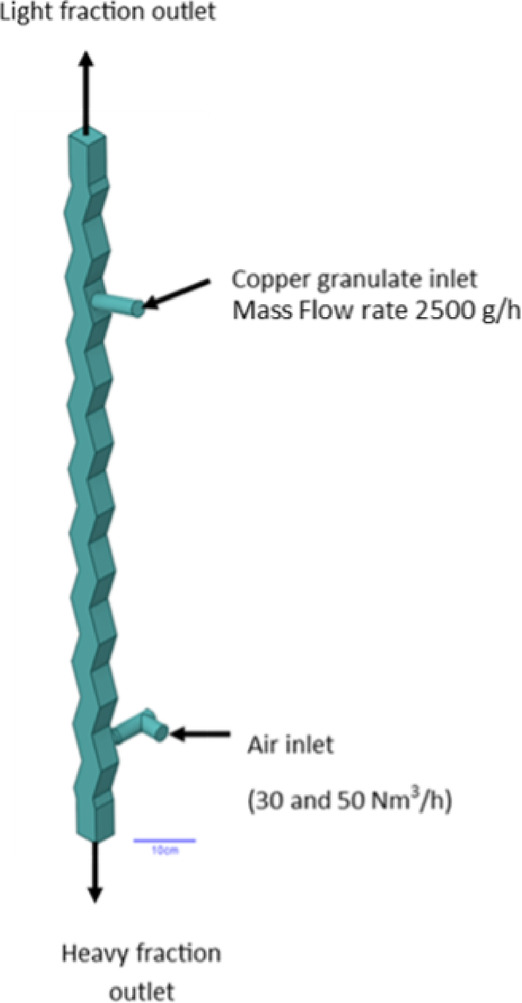
Graphical representation of the model boundary conditions.

Simulations were performed using ANSYS Fluent 22R2 software. The boundary conditions for the simulations were as follows:

- airflow rate for granulate separation: 30 and 50 Nm³/h,
- granulate mass flow rate fed into the separator working space: 2500 g/s (Fig. 10).

The simulations were carried out under steady-state conditions. The k-epsilon turbulence model was employed, incorporating Standard Wall Functions for boundary layer interactions. The behavior of solid copper particles (granules) was simulated using the Discrete Phase Model (DPM), accounting for particle non-sphericity (*ψ*).

The particle size distribution used in the presented simulations was determined based on the sieve analysis of the tested material. Based on the conducted sieve analyses (Table 6), the average sphericity of the granulate was established at *ψ* = 0,23.

**Table 6:** Particle size distribution of copper granules for the investigated samples.

Fraction size [µm]	Mass fraction of particles [%_mass_]			
	Granulate 1	Granulate 2	Granulate 3	Granulate 4
x < 71	0	0.05	0.01	0.07
71 < x < 125	0.76	0.72	0.05	0.03
125 > x > 315	17.42	7.09	1.22	12.91
315 < x < 500	17.78	14.71	9.17	20.10
500 < x < 1000	31.88	15.70	15.09	26.82
1000 < x < 2000	31.67	59.29	67.22	35.89
2000 < x < 3000	0.48	2.44	7.17	3.66
3000 < x < 5000	0.01	0	0.07	0.51
5000 < x	0	0	0	0.01

The mass flow rate of the particles and the volumetric flow rate of the air were determined during the experimental phase. During this phase, a research range of 30–50 Nm³/h for air and a feed rate of 500 g/h for the solid particles delivered to the separator were established. The adopted convergence criteria are presented in Table 7.

**Table 7:** Convergence criteria applied in the simulation.

Continuity	0.0001
x-velocity	0.0001
y-velocity	0.0001
z-velocity	0.0001
K	0.0001
epsilon	0.0001

The simulations were conducted for two airflow rates (30 and 50 Nm³/h) and four types of granulates (obtained from the processing of various electrical cables in an industrial plant), each characterized by a different chemical composition. The chemical composition of these materials is detailed in Table 8.

**Table 8:** Chemical composition of the investigated granulate samples.

Component	Granulate 1	Granulate 2	Granulate 3	Granulate 4
	%			
Tin (Sn)	0.110	0.018	0.020	0.068
Zinc (Zn)	0.051	0.37	0.230	0.300
Aluminium (Al)	0.032	0.21	0.250	0.150
Lead (Pb)	0.005	0.022	0.360	0.039
Iron (Fe)	0.019	0.11	0.015	0.200
Copper (Cu)	99.78	99.27	99.11	99.21

## Results and discussion

**Fig. 11:**
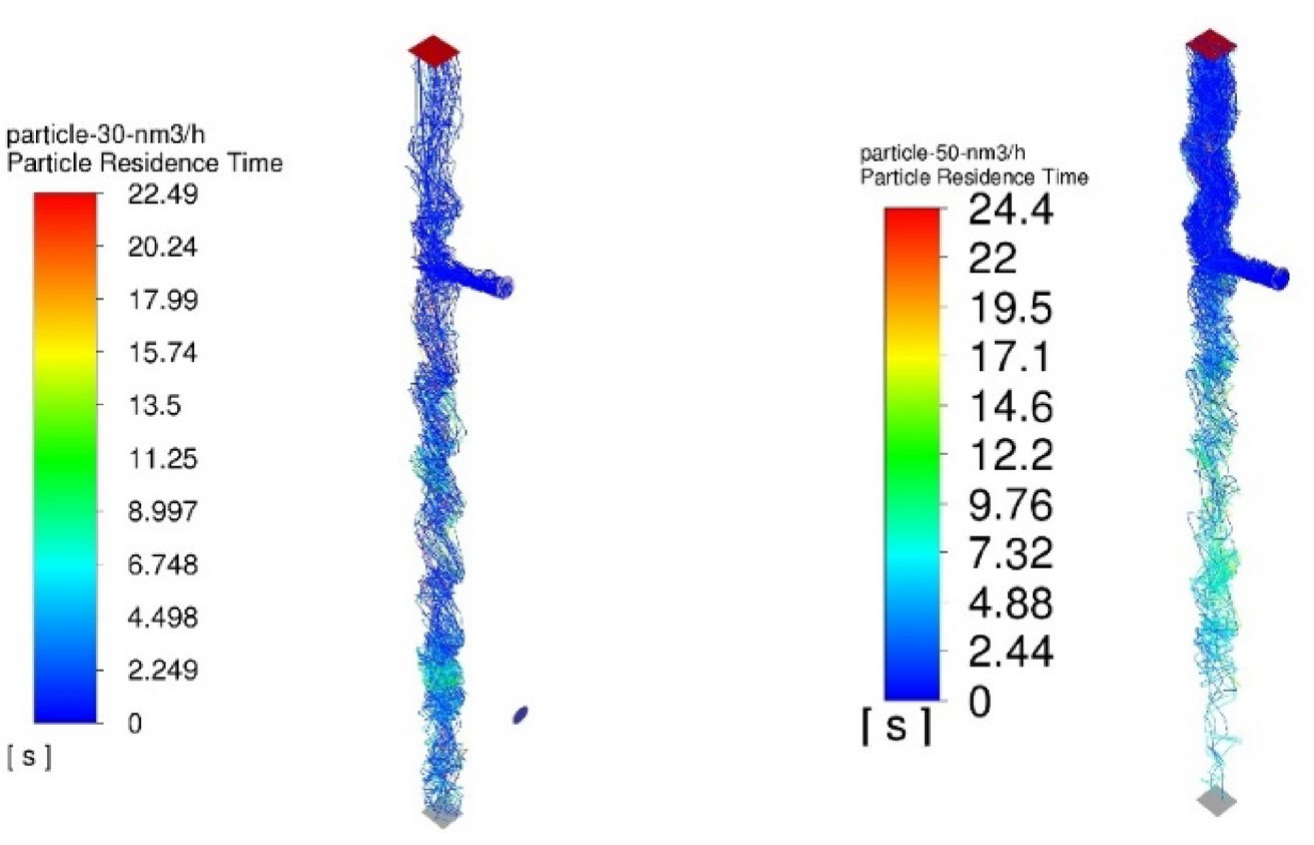
Particle trajectories for pure copper (heavy fraction) and tinned copper (light fraction) for Granulate 1 (left – 30 Nm^3^/h; right– 50 Nm^3^/h).

**Fig. 12:**
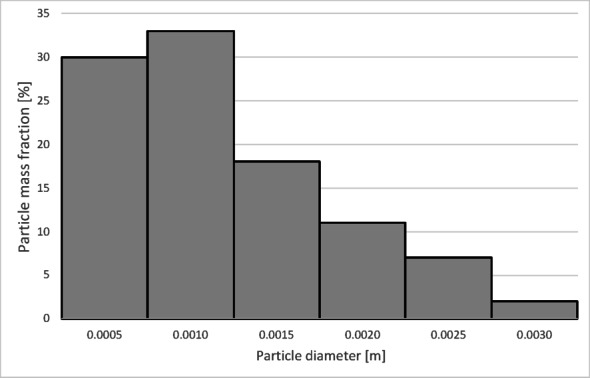
Particle size distribution histogram for the light fraction of Granulate 1 (air flow rate of 30 Nm^3^/h).

**Fig. 13:**
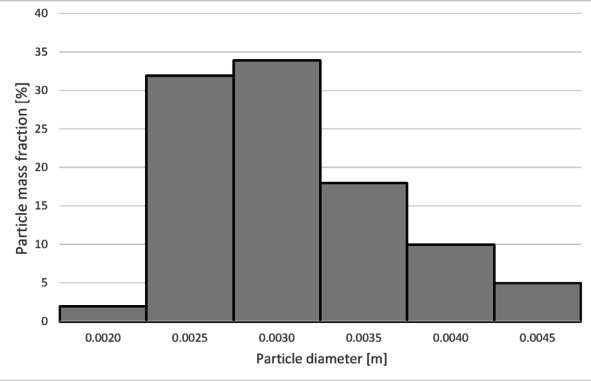
Particle size distribution histogram for the heavy fraction of the Granulate 1 (air flow rate of 30 Nm^3^/h).

**Fig. 14:**
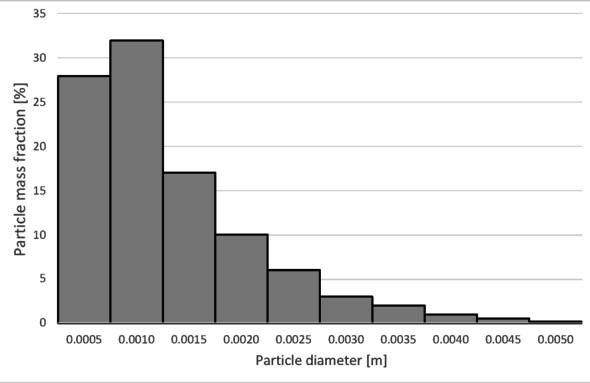
Particle size distribution histogram for the light fraction of Granulate 1 (air flow rate of 50 Nm3/h).

**Fig. 15:**
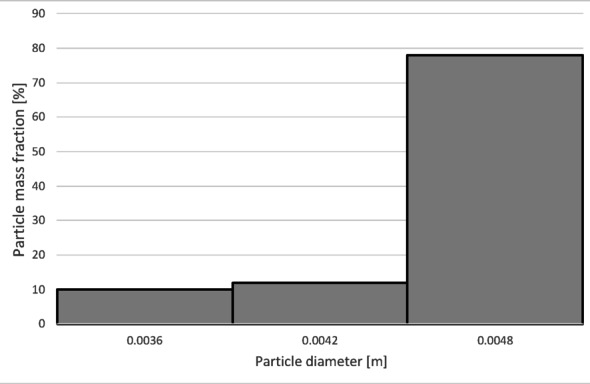
Particle size distribution histogram for the heavy fraction of the Granulate 1 (air flow rate of 50 Nm3/h).

The numerical results of the particle trajectory calculations for Granulate 1 are presented in Figs. 11, 12, 13, 14 and 15.

During the separation calculations for Granulate 1 at an airflow rate of 30 Nm³/h, a smaller proportion of particle trajectories directed toward the upper outlet (forming the light fraction – Fig. 11) is observed. The average residence time of particles in the separator’s working zone is approximately 9 s.

Increasing the airflow to 50 Nm³/h leads to an increase in the mass of the light fraction and the removal of larger diameter particles from the total separated material stream (Fig. 11). There is also an increase in the average particle residence time to 12 s. This is caused by a greater buoyancy [drag] force; consequently, larger particles are entrained higher into the separator’s working space by the air stream before eventually falling to the bottom outlet.

To determine the separation efficiency, histograms showing the distribution of particles collected at each separator outlet were prepared (Figs. 12, 13, 14 and 15).

From the histograms presented in Figs. 12 and 13, it can be observed that the light fraction consists of particles smaller than 3 mm, with particles up to 1 mm representing the largest share (totaling 63%). It is also noted that the heavy fraction predominantly comprises particles larger than 2.3 mm, with those in the 2.7 to 3.6 mm range constituting the largest portion (approximately 64% in total).

Increasing the airflow rate to 50 Nm³/h leads to the appearance of particles sized 3 to 5 mm in the light fraction; however, their share remains at a level of 5–6% of the total mass of this fraction (Fig. 14). Particles ranging from 3.3 mm to 5 mm pass into the heavy fraction (Fig. 15), where particles sized 4.5 to 5 mm are the most prevalent (79%).

**Fig. 16:**
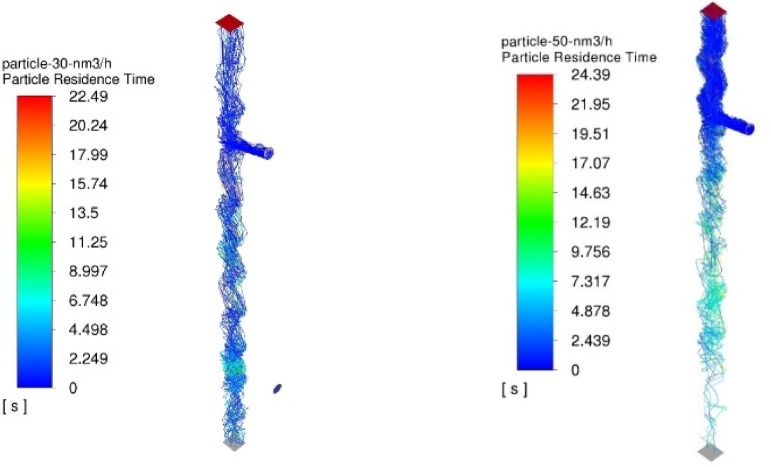
Particle trajectories for pure copper (heavy fraction) and tinned copper (light fraction) for Granulate 2 (left – 30 Nm^3^/h; right– 50 Nm^3^/h).

**Fig. 17:**
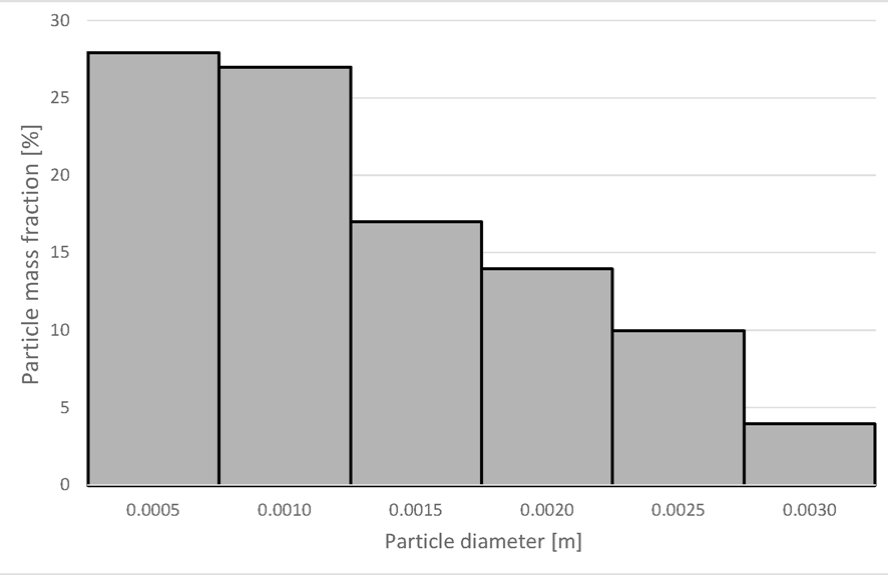
Particle size distribution histogram for the light fraction of Granulate 2 (air flow rate of 30 Nm^3^/h).

**Fig. 18:**
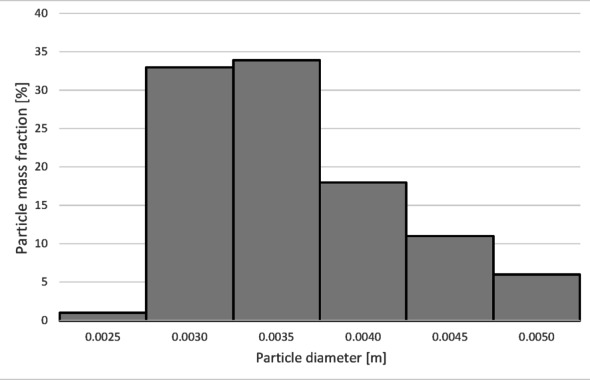
Particle size distribution histogram for the heavy fraction of the Granulate 2 (air flow rate of 30 Nm^3^/h).

**Fig. 19:**
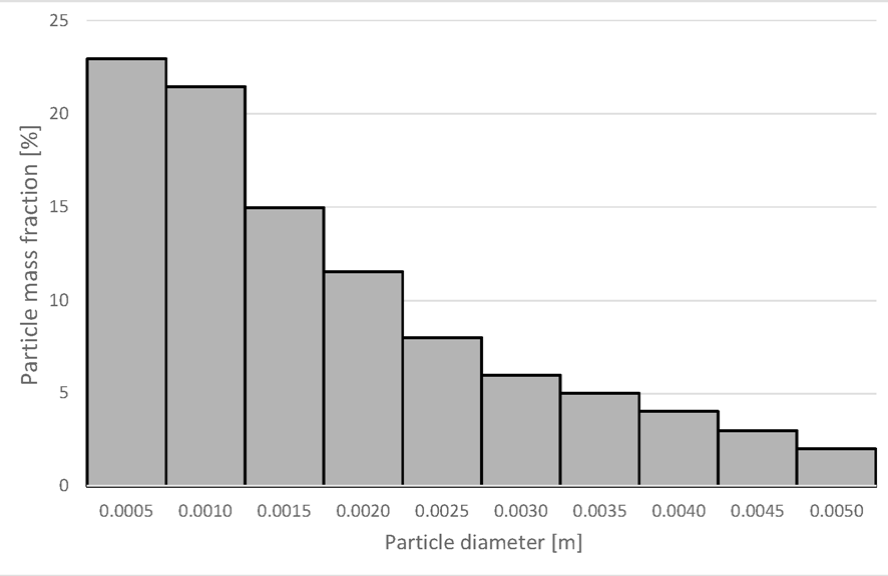
Particle size distribution histogram for the light fraction of Granulate 2 (air flow rate of 50 Nm^3^/h).

**Fig. 20:**
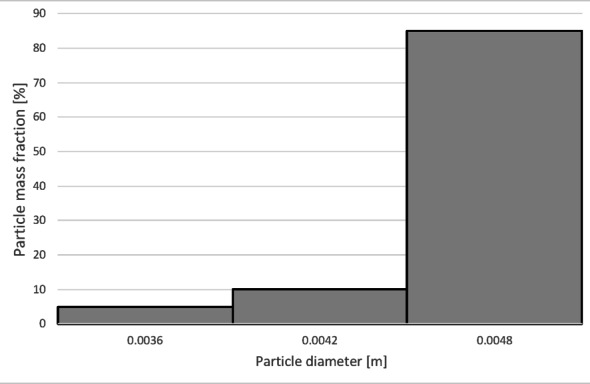
Particle size distribution histogram for the heavy fraction of the Granulate 2 (air flow rate of 50 Nm^3^/h).

The numerical results of the particle trajectory calculations for Granulate 2 are presented in Figs. 16, 17, 18, 19 and 20.

Conducting the process with an airflow rate of 30 Nm³/h results in a uniform distribution of particles between the two fractions (Fig. 16). The average residence time of the particles in the separator’s working zone is approximately 9 s.

Increasing the separation airflow rate to 50 Nm³/h further increases the mass flow of material into the light fraction. This is accompanied by an increase in the average particle residence time to 10 s (as compared to the results obtained for the 30 Nm³/h airflow rate).

The histograms presented in Figs. 17 and 18 show the particle size distribution of the copper granulate forming the heavy and light fractions at a flow rate of 30 Nm³/h. It can be observed that only a small amount of 2.5 mm particles is retained in the heavy fraction (representing approximately 1% of this fraction). This fraction predominantly consists of particles ranging from 2.7 to 5 mm (99% share). Conversely, particles up to 3 mm in size are found in the light fraction.

Further increasing the airflow rate to 50 Nm³/h leads to the removal of particles with diameters approximately 0.1 mm larger from the heavy fraction (Fig. 20). In the light fraction, a small number of particles with diameters exceeding 4 mm is observed (Fig. 19).

**Fig. 21:**
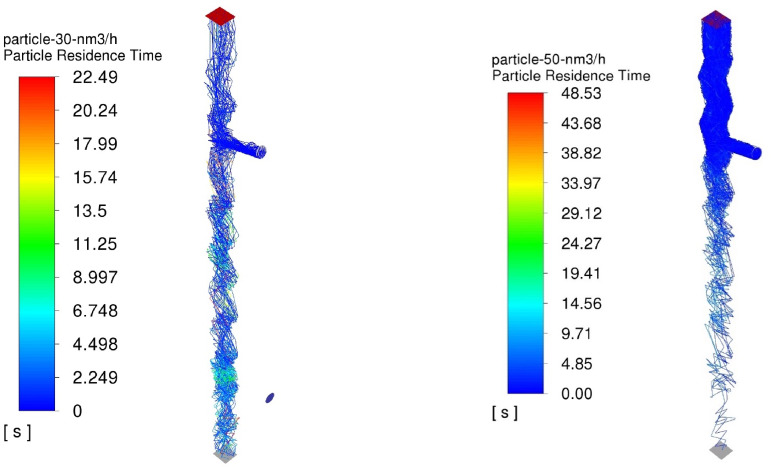
Particle trajectories for pure copper (heavy fraction) and tinned copper (light fraction) for Granulate 3 (left – 30 Nm^3^/h; right– 50 Nm^3^/h).

**Fig. 22:**
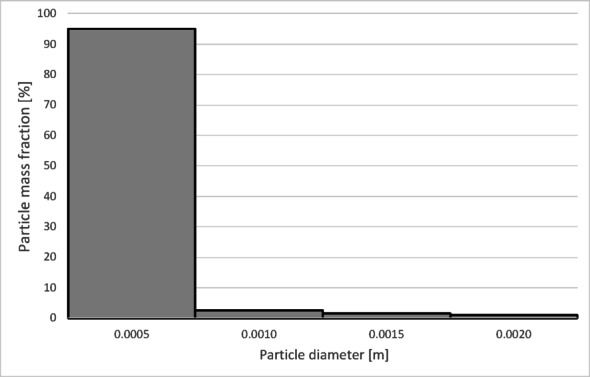
Particle size distribution histogram for the light fraction of Granulate 3 (air flow rate of 30 Nm^3^/h).

**Fig. 23:**
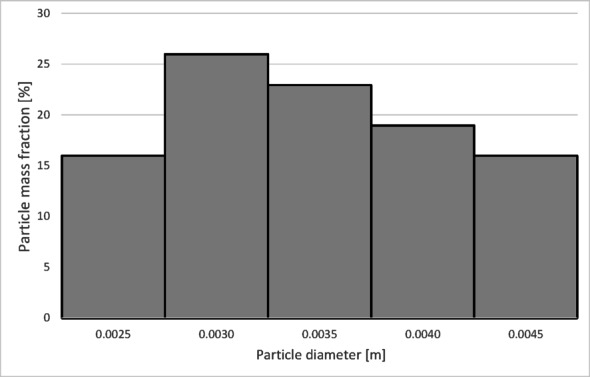
Particle size distribution histogram for the heavy fraction of the Granulate 3 (air flow rate of 30 Nm^3^/h).

**Fig. 24:**
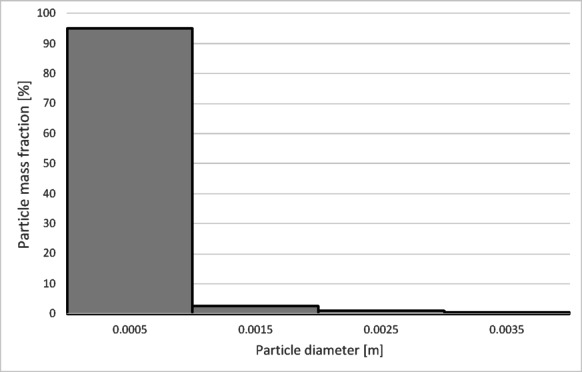
Particle size distribution histogram for the light fraction of Granulate 3 (air flow rate of 50 Nm^3^/h).

**Fig. 25:**
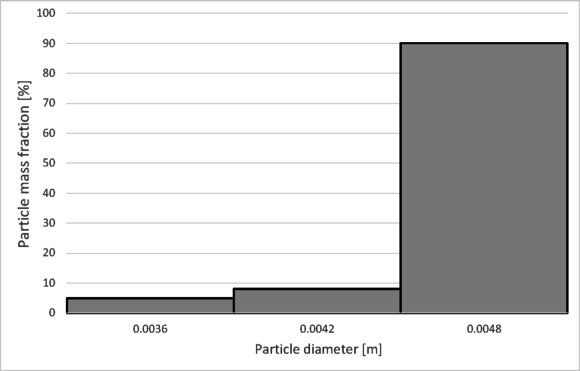
Particle size distribution histogram for the heavy fraction of the Granulate 3 (air flow rate of 50 Nm^3^/h).

The numerical results of the particle trajectory calculations for Granulate 3 are presented in Figs. 21, 22, 23, 24 and 25.

At an airflow rate of 30 Nm³/h, it is observed that the particles are distributed uniformly between the light and heavy fractions. For this flow rate, the average particle residence time in the separator’s working zone is approximately 7 s. Increasing the airflow to 50 Nm³/h leads to a nearly complete transition of the material into the light fraction. This indicates a much more intense entrainment of light particles and their migration into the light fraction. This is further confirmed by the increase in the average particle residence time in the separator’s working space to approximately 14 s.

Analyzing the histogram in Fig. 22, it can be observed that over 95% of the granules passing into the light fraction are particles smaller than 0.5 mm. Particles ranging from 0.5 to 3.35 mm account for approximately 5% of the total amount of copper granules in the light fraction. Particles larger than 2 mm pass into the heavy fraction (Fig. 23).

As the airflow increases to 50 Nm³/h, the diameter of particles migrating to the light fraction increases up to 5 mm; however, approximately 95% of the light fraction still consists of granules up to 0.5 mm (Fig. 24).

Simultaneously, the diameter of particles passing into the heavy fraction shifts toward larger sizes, with particles from 3.2 mm upward predominantly being collected there (Fig. 25). This indicates an improvement in the separation of smaller-diameter particles containing impurities (tin) from larger particles (primarily pure copper granules).

**Fig. 26:**
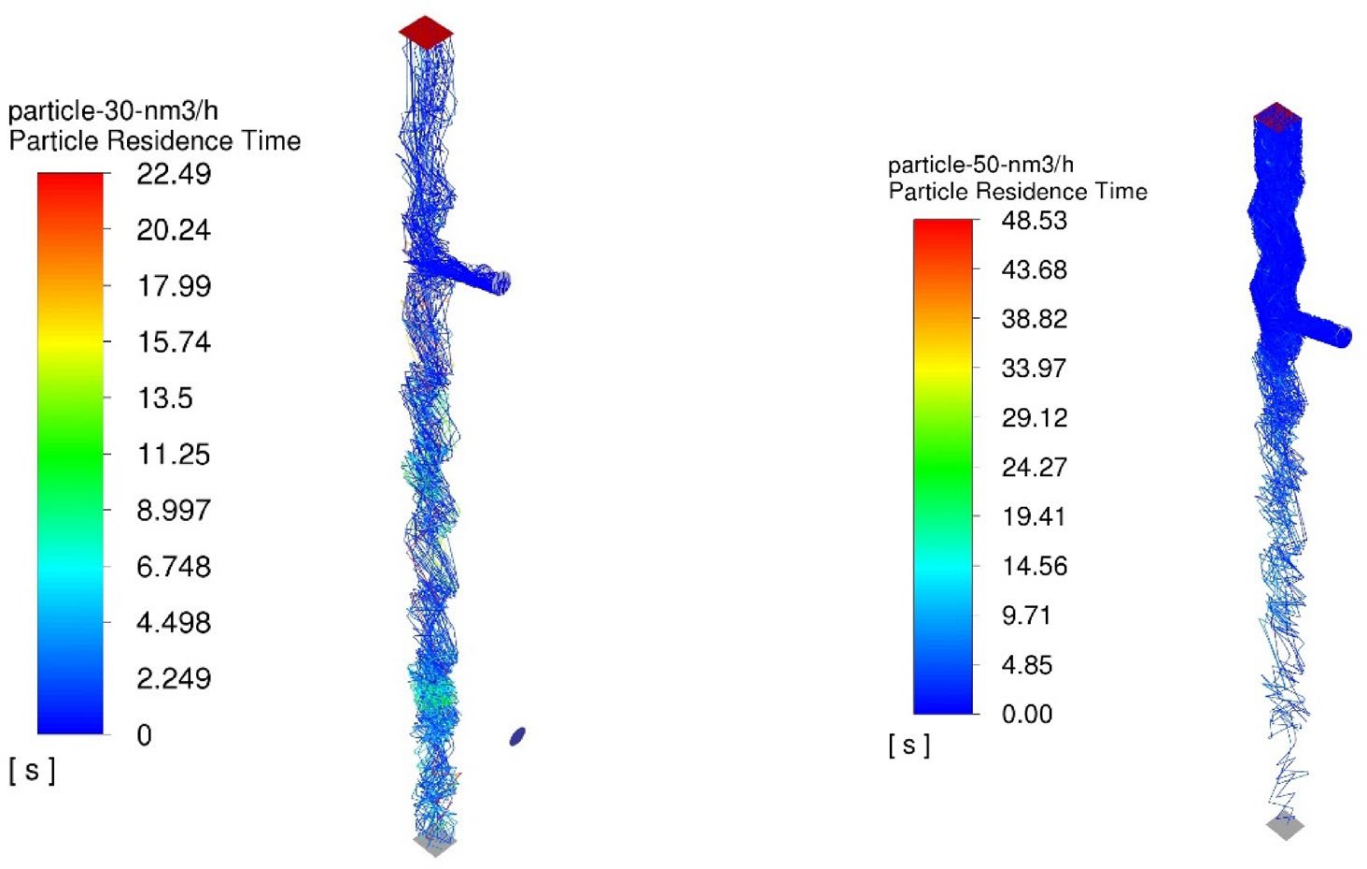
Particle trajectories for pure copper (heavy fraction) and tinned copper (light fraction) for Granulate 4 (left – 30 Nm^3^/h; right– 50 Nm^3^/h).

**Fig. 27:**
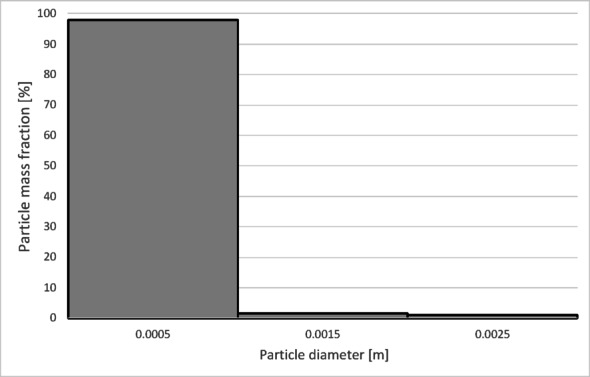
Particle size distribution histogram for the light fraction of Granulate 4 (air flow rate of 30 Nm^3^/h).

**Fig. 28:**
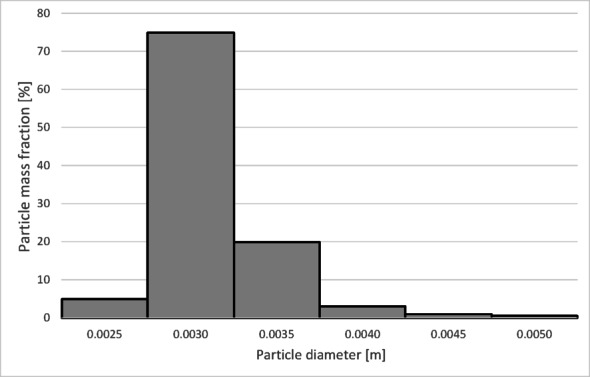
Particle size distribution histogram for the heavy fraction of the Granulate 4 (air flow rate of 30 Nm^3^/h).

**Fig. 29:**
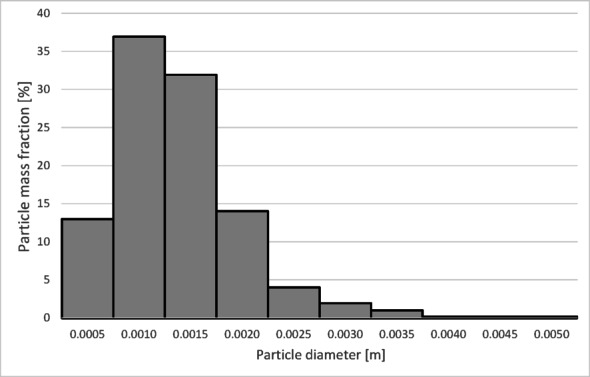
Particle size distribution histogram for the light fraction of Granulate 4 (air flow rate of 50 Nm^3^/h).

**Fig. 30:**
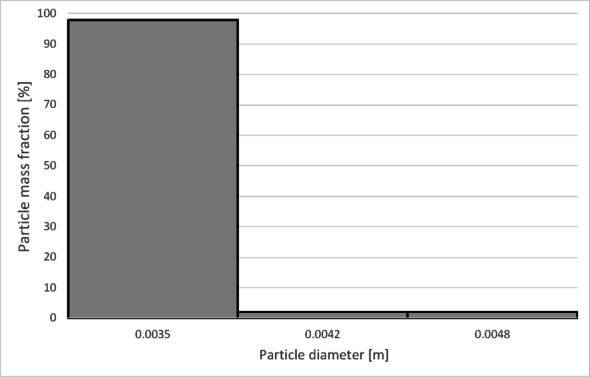
Particle size distribution histogram for the heavy fraction of the Granulate 4 (air flow rate of 50 Nm^3^/h).

The numerical results of the particle trajectory calculations for Granulate 4 are presented in Figs. 26, 27, 28, 29 and 30.

Analyzing the particle trajectories at a flow rate of 30 Nm³/h, it can be observed that the particles are distributed uniformly between both fractions. This may result in a low degree of separation between copper particles and tinned elements. The average particle residence time for this airflow rate is 7 s. Increasing the separation airflow to 50 Nm³/h leads to a reduction in the number of particles reaching the heavy fraction and increases the residence time in the separator’s working zone to approximately 10 s.

At an airflow rate of 30 Nm³/h, particles larger than 2 mm are collected in the heavy fraction (Fig. 28). However, the largest share of the heavy fraction consists of particles sized between 3 and 3.2 mm, accounting for approximately 74% of its mass. Conversely, the light fraction primarily contains particles up to 1 mm in diameter (Fig. 27), representing about 95% of the total product mass. Larger particles (1 to 5 mm) also reach this fraction, but their combined share does not exceed 5%.

Increasing the airflow to 50 Nm³/h increases the content of particles larger than 1 mm in the light fraction (Fig. 29). The weight distribution of various particle sizes within the total product mass also changes; while particles up to 1 mm remain the most numerous, their share drops to approximately 50%. A significant presence of 1 to 2 mm particles is observed, at approximately 31% and 14%, respectively. Particles sized 2 to 3 mm are also noticeable. Consequently, only larger particles, starting from 2.7 mm, pass into the heavy fraction (Fig. 30). In this case, the heavy fraction is composed of particles with diameters ranging from 3.3 to 3.9 mm, which together account for about 98% of the fraction.

The modeling results were compared with the experimental data. Based on this comparison, the model error was determined (Table 9) according to the following formula:

$$Error = \left[ \left( \frac{\text{value calculated from the model}}{\text{heavy fraction mass}} \right) \times 100\% \right] - 100$$

A negative error value indicates that the result obtained from numerical calculations (the model) is lower than the result obtained from laboratory experiments.

**Table 9:** Comparison of heavy and light fraction yields: experimental results vs. numerical analysis.

	Granulate 1		Granulate 2		Granulate 3		Granulate 4	
	Fraction mass		Fraction mass		Fraction mass		Fraction mass	
	heavy	light	heavy	light	heavy	light	heavy	light
	Airflow rate 30 Nm^3^/h							
Experimental value [%]	39.7	60.3	76.93	23.04	88.21	11.75	43.01	56.82
Calculated value [%]	28.24	71.76	76.77	23.23	95.34	4.66	45.40	54.60
Relative error [%]	-28.86	19.00	-0.20	0.81	8.09	-60.36	5.56	-3.91
	Airflow rate 50 Nm^3^/h							
Experimental value [%]	22.17	77.95	56.08	43.95	54.24	45.81	2.09	97.8
Calculated value [%]	16.17	83.83	52.32	47.68	2.90	97.10	0.72	99.28
Relative error [%]	-27.08	7.55	-6.71	8.50	-94.66	111.97	-65.70	1.52

The results presented in Table 9 show that the error between the model and the actual experimental results depends on the type of granulate tested. The best convergence between the model and experimental data was observed for Granulate 2, with an error ranging from 0.2% to 8.5%. The model exhibited the lowest convergence for Granulate 3, where the error ranged from 8.09% to 111.97%. It should also be noted that increasing the separation airflow rate from 30 to 50 Nm³/h leads to an increase in the error values.

For Granulates 1 and 4, the model error (relative to experimental results) ranged from 1.52% to 67.50%. In these cases, increasing the airflow rate from 30 to 50 Nm³/h generally improved the model’s accuracy, with the exception of the heavy fraction for Granulate 4, where a ten-fold increase in error was observed.

The model’s inaccuracy cannot be entirely attributed to differences in the grain size distribution of the individual granulates. Granulate 2 (which yielded the best convergence) and Granulate 3 (which yielded the lowest) possess very similar sieve compositions. Both granulates primarily consist of fractions: 0.315–0.5 mm (Granulate 2: 14.71%; Granulate 3: 9.17%), 0.5–1 mm (Granulate 2: 15.70%; Granulate 3: 15.09%), and 1–2 mm (Granulate 2: 59.29%; Granulate 3: 67.22%). Therefore, the differences in error values must be caused by the sphericity coefficient, which was set at. *ψ* = 0.23 for all granulates.

A sphericity value far from 1 indicates how much the actual shape deviates from a sphere. The lower the value, the more the calculations tend to diverge from the real values. Consequently, the sphericity coefficient must be determined individually for each specific material. This represents the greatest challenge in scaling laboratory results to industrial equipment, as it directly impacts separation parameters and, ultimately, the quality of the final product.

## Conclusions

The results of the conducted research demonstrate that the zig-zag air classifier can effectively increase the purity of copper granulates obtained from electrical cable recycling lines. The use of the separator allows for an increase in copper content in the granulate up to 99.88% Cu (Granulate 2, Tables 2, 50 Nm³/h).

The developed numerical model of the air separation process in the zig-zag classifier enables the determination of the distribution of individual separation fractions (heavy and light). The error value between the modeling results and the actual experimental data is influenced by the morphology of the tested granulate and the shape of the granules themselves. The determined model error ranged from 0.20% (Granulate 1, Table 9) to as high as 111.97% (Granulate 4, Table 9). To reduce the model error, the sphericity coefficient must be determined individually for each analyzed granulate.

## Data Availability

The datasets generated and/or analysed during the current study are not publicly available as these data belong to the Lukasiewicz Research Network - Institute of Non-ferrous Metals, but are available from the author responding to a reasonable request after obtaining permission from the management of the Lukasiewicz Research Network - Institute of Non-ferrous Metals. Requests for data sharing should be addressed to Mr. Grzegorz Krawiec at: grzegorz.krawiec@imn.lukasiewicz.gov.pl.

## References

[1] https://www.visualcapitalist.com/sp/bhp01-global-copper-demand-2021-2050p/

[2] https://invest.conotoxia.com/investment-research/comments/will-the-copper-price-rise-what-is-driving-the-copper-price-in-the-markets

[3] https://www.cire.pl/artykuly/energetyka/186609-miedz-ekologiczny-metal-przyszlosci

[4] Guzik, K., Galos, K. & Czerw, H. *„Wykorzystanie surowców mineralnych w gospodarce i życiu codziennym człowieka* (IGSMiE PAN, 2023).

[5] https://elektryczny.pl/elektroenergetyczne-k42030108

[6] https://strefainzyniera.pl/artykul/1199/pojecie-i-klasyfikacja-kabli-elektrycznych

[7] https://praktycznie-elektryka.blogspot.com/2017/09/teoria-o-kablach-i-przewodach.html

[8] https://www.rynekelektryczny.pl/

[9] Pita i, F. & Castilho, A. „Separation of Copper from Electric Cable Waste Based on Mineral Processing Methods: A Case Study. *Minerals* **8** (517). (2018). 10.3390/min8110517

[10] Park, C. H., Subasinghe, N. & Jeon, H. S. „Separation of Covering Plastics from Particulate Copper in Cable Wastes by Induction Electrostatic Separation, MATERIALS TRANSACTIONS,56, (2015). 10.2320/matertrans.M2015138

[11] Catinean, A., Dascalescu, L., Lungu, M., Dumitran, L. M. & Samuila, A. Improving the recovery of copper from electric cable waste derived from automotive industry by corona-electrostatic separation, *Part. Sci. Technol.*, **39**, 1–8, (2020). 10.1080/02726351.2020.1756545

[12] Katsari i, C. M. & Anastassakis, G. „Copper recovery from electric cable scrap by shaking tables, zaprezentowano na XXVIII International Mineral Processing Congress (IMPC 2016).

[13] Yokoyama, S. & Takeuchi, S. i N. Hisyamudin Bin Muhd Nor, „Mechanical Separation of Metallic Copper from Polymer-Insulated Copper Wire, *AIP Conf. Proc.*, **1315**(1), 1527–1532, (2011). 10.1063/1.3552405

[14] Stepanenko, S., Kotov, B., Kuzmych, A., Kalinichenko, R. & Hryshchenko, V. Research of the process of air separation of grain material in a vertical zigzag channel. *J. Cent. Eur. Agric.* **24** (1), 225–235. (2023). 10.5513/JCEA01/24.1.3732

[15] Bala, B. K. *Agro-Product Processing Technology: Principles and Practice* (CRC, 2020). 10.1201/9780429487507

[16] White, F. M. *Fluid Mechanics, 7. wyd* (McGraw Hill, 2009).

[17] Blazek, J. *Computational Fluid Dynamics: Principles And Applications* (Elsevier Science Ltd, ).

[18] ANSYS Fluent 2022R2 Theory Guide. ANSYS, Inc., (2022).

[19] Zhang, J., Wang, L. & Ouyang, J. „Lattice Boltzmann Model for The Volume-Averaged Navier-Stokes Equations. *EPL* **107** (2), 20001. (2014). 10.1209/0295-5075/107/20001

[20] Blais, B., Tucny, J. M., Vidal, D. & Bertrand, F. A conservative lattice Boltzmann model for the volume-averaged Navier–Stokes equations based on a novel collision operator. *J. Comput. Phys.* **294**, 258–273. (2015). 10.1016/j.jcp.2015.03.036

[21] „Wu, C. L., Nandakumar, K., Berrouk, A. S. & Kruggel-Emden, H. Enforcing mass conservation in DPM-CFD models of dense particulate flows. *Chem. Eng. J.* **174**, 475–481 (2011). - Szukaj w Google. Dostęp: 9 maja 2025. [Online].

[22] Wu, C. L., Berrouk, A. S. & Nandakumar, K. „Three-dimensional discrete particle model for gas–solid fluidized beds on unstructured mesh. *Chem. Eng. J.* **152** (2), 514–529. (2009). 10.1016/j.cej.2009.05.024

[23] Wu, C. L., Zhan, J. M., Li, Y. S., Lam, K. S. & Berrouk, A. S. „Accurate void fraction calculation for three-dimensional discrete particle model on unstructured mesh. *Chem. Eng. Sci.* **64** (6), 1260–1266. (2009). 10.1016/j.ces.2008.11.014

